# Comparative Study on Crack Initiation and Propagation of Glass under Thermal Loading

**DOI:** 10.3390/ma9100794

**Published:** 2016-09-22

**Authors:** Yu Wang, Qingsong Wang, Haodong Chen, Jinhua Sun, Linghui He

**Affiliations:** State Key Laboratory of Fire Science, University of Science and Technology of China, Hefei 230026, China; ywang232@mail.ustc.edu.cn (Y.W.); linghao@mail.ustc.edu.cn (H.C.); sunjh@ustc.edu.cn (J.S.); lhhe@ustc.edu.cn (L.H.)

**Keywords:** glass, finite element method, thermal stress, crack initiation, crack growth

## Abstract

This paper explores the fracture process based on finite element simulation. Both probabilistic and deterministic methods are employed to model crack initiation, and several commonly used criteria are utilized to predict crack growth. It is concluded that the criteria of maximum tensile stress, maximum normal stress, and maximum Mises stress, as well as the Coulomb-Mohr criterion are able to predict the initiation of the first crack. The mixed-mode criteria based on the stress intensity factor (SIF), energy release rate, and the maximum principal stress, as well as the SIF-based maximum circumferential stress criterion are suitable to predict the crack propagation.

## 1. Introduction

Windows are among the weakest parts in a building and have drawn great attention when considering the case of fire. Upon heating, a window glass may break and release the accumulated thermal stress. This opens a gate for oxygen flow from the outside into the room, and thus accelerates the fire development. The breakage of glass exposed to fire includes two stages. First, cracks initiate and propagate in a glass that is under heating. Then they coalesce to form “islands”, followed by the falling out of the fragments. Since this process may exert considerable influence on the development of a fire, understanding this mechanism is of important significance not only for fundamental research but also for practical applications.

The edges of a window glass are covered by a frame. When exposed to a fire, the temperatures in the central portion and edge region are different. It is believed that the glass cracks when this temperature difference reaches a critical value. However, the data of the critical temperature difference proposed by different authors exhibit considerable discrepancy. For example, the theoretical predictions given by Keski-Rahkonen [1] and Pagni and Joshi [2,3,4] are 80 °C and 58 °C, respectively, while the experimental measurements reported by Skelly et al. [5] and Shields et al. [6] vary from 90 to 150 °C, respectively. Klassen et al. [7] examined the effect of sample size on the temperature difference for crack initiation, and found that the critical values for small (305 × 305 mm^2^) and medium (609 × 1219 mm^2^) samples are less than 200 °C and 300 °C, respectively.

The non-uniform temperature distribution in a glass exposed to fire may be influenced by various factors, such as the thermal and mechanical properties of the glass, the decay length of flame radiation, and the heating methods [3,4,5]. In particular, the presence of surface flaws can significantly reduce the practical strength of a glass product [8]. These factors obviously endow some probabilistic characteristics to crack initiation in glass [9]. For this reason, Joshi and Pagni [4] proposed a three-parameter cumulative Weibull function that can be used to model the fracture of ordinary window glass, and Hietaniemi [9] developed a probabilistic approach to evaluate the breakage condition for a window pane heated by a fire. Nonetheless, research in this direction is still under way, and the current capability of predicting crack initiation and propagation in glass can and should be largely enhanced. This is the motivation of the present study. Based on a finite element simulation, this paper explores the fracture process of a window glass under the thermal loading of fire. Both the probabilistic and deterministic methods are adopted, and the main objective is to examine the validities of some commonly used criteria of crack initiation and propagation for window glasses under fire conditions.

## 2. Thermal Stress Distribution

**Figure 1:**
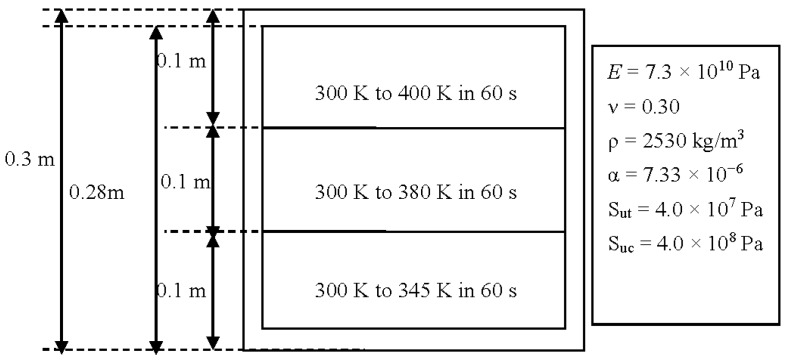
Problem statement of the glass under thermal loading. The glass properties are also reported ([10,11,12,13]).

**Figure 2:**
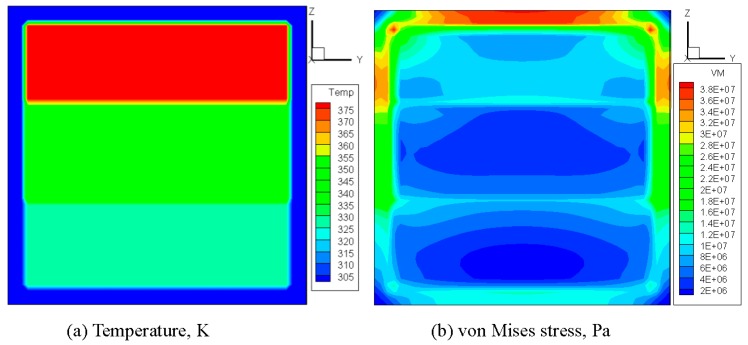
Distributions of temperature and von Mises stress in the glass before crack initiation. (**a**) Temperature distribution in glazing; (**b**) Von Mises Stress. (“3.8E+07” represents “3.8 × 10^7^” in legend).

In the case of a fire, window glass is normally subjected to a non-uniform heating, due to the fact that the temperature of the upper portion is higher than that of the lower portion. To investigate the glazing behavior, a simple assumption of a three layer rising temperature was adopted. It is the authors’ belief that although this assumption is not the same as a real fire, it is suitable for the investigation in the present work, as the objective is to investigate different criteria and determine some suitable methods to predict crack initiation and propagation. A square glass pane with a dimension of 0.003 × 0.3 × 0.3 m^3^ was used in the simulation. As shown in Figure 1, the edge of the glass pane is covered by a frame with a width of 0.02 m. The central portion of the pane is divided vertically into three regions in which the heating rates are different. The sample was heated at 300 K for 60 s, and the final temperatures in the upper, middle, and lower regions were 400 K, 380 K, and 345 K, respectively. At the edge, the glass is shaded by the window frame, so the glass rising temperature is quite small in a real fire. Therefore, the glass edge temperature is assumed to be invariant. Using an in house software, the thermal stress in the glass was computed via 45 × 45 × 2 hexahedron elements [10], with material constants cited from previous studies [11,12,13,14]. It is noted that some glass parameters are temperature-dependent [15], but in the present simulation the temperature range is very small and when the crack occurs, the maximum temperature in glazing is around 380 K. In such a small temperature range, the variance of material parameters is very limited [16] and is thus ignored in the simulation. Figure 2a,b reports the calculated distributions of the temperature and von Mises stress, respectively. It can be seen that the von Mises stress attains its maximum value in the central part of the upper edge. All the other stress components, including the principal stress values *σ*_1_, *σ*_2_, and *σ*_3_ are obtainable. The results are used as a starting point in the subsequent analysis of crack initiation.

## 3. Modeling of Crack Initiation

As the glass crack initiation shows some stochastic behaviour, a probabilistic criterion is used to predict the glass crack initiation. For comparison, another deterministic criterion is employed in the simulation to predict when and where a crack initiates in the glass pane under the thermal conditions.

### 3.1. Probabilistic Criterion

Based on their experiments of the four-point bending test for 59 glass plates, Joshi and Pagni [4] described the distribution of the breaking stress by a three-parameter cumulative Weibull function as follows:

(1) $$F(\sigma_{b})=\left\{ \begin{array}{ll} 0 & \;\sigma_{b}<\sigma_{u}\\ 1-\exp[-{\left(\frac{\sigma_{b}-\sigma_{u}}{\sigma_{0}}\right)}^{m}] & \sigma_{b}\geq \sigma_{u} \end{array}\right.$$

where *σ*_b_ is the breaking stress, *σ*_0_ and *m* are the scale and shape parameters in the Weibull distribution function, and *σ*_u_ is often referred to as the zero strength or lowest strength of the specimen. The values of *m*, *σ*_0_, and *σ*_u_ were obtained by data fitting and the detailed values are listed in Table 1 [4]. Further experiments by Pagni [17] with different glass thicknesses enable one to assess the dependence of these parameters on the glass thickness *L*. The equations characterizing this dependence, written in a non-dimensional form, are as follows [9,17]:

(2) $$\frac{\sigma_{u}}{\mathrm{MPa}}=0.5016(\frac{L}{\mathrm{mm}})+34.59$$

(3) $$\frac{\sigma_{0}}{\mathrm{MPa}}=-0.2919{\left(\frac{L}{\mathrm{mm}}\right)}^{2}+2.9309(\frac{L}{\mathrm{mm}})+27.497$$

(4) $$m=0.000815{\left(\frac{L}{\mathrm{mm}}\right)}^{2}-0.000985(\frac{L}{\mathrm{mm}})+1.205$$

#### 3.1.1. Two-Parameter Weibull Distribution

**Figure 3:**
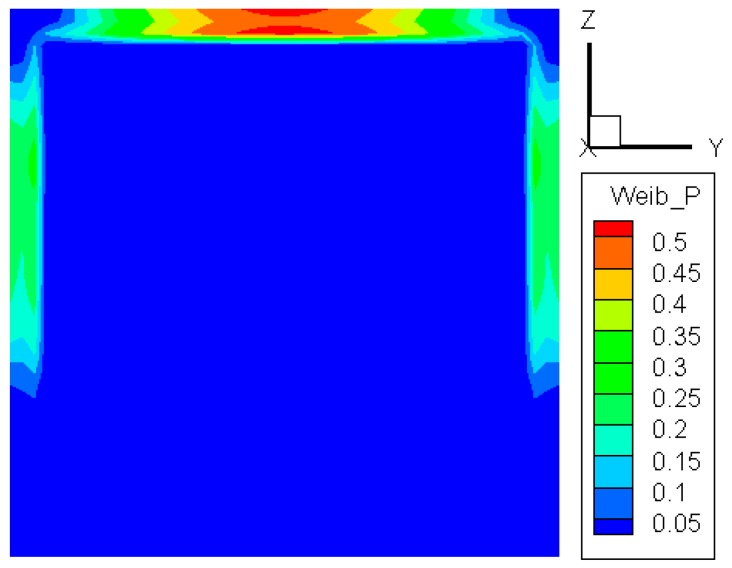
Probability distribution of crack initiation via the two-parameter Weibull distribution.

When *σ*_u_ is taken as zero, Equation (1) becomes the two-parameter Weibull distribution. In this case, the probability distribution of crack initiation in the glass is shown in Figure 3. It can be noted that the probability is higher in the edge region than in the central portion of the pane. The highest probability occurs in the portion near the central part of the upper edge, where both the temperature and tensile stress attain their maximum values. At the two side edges, the probability becomes smaller. The smallest probability appears at the lower edge. The Weibull method can predict the probability distribution of glass crack initiation, and the maximum value is selected here. In our simulations, we postulate that the glass cracks if the probability is larger than 0.5. As to the random crack initiation location, this needs another model for its prediction [18], which is not discussed here.

#### 3.1.2. Three-Parameter Weibull Distribution

**Figure 4:**
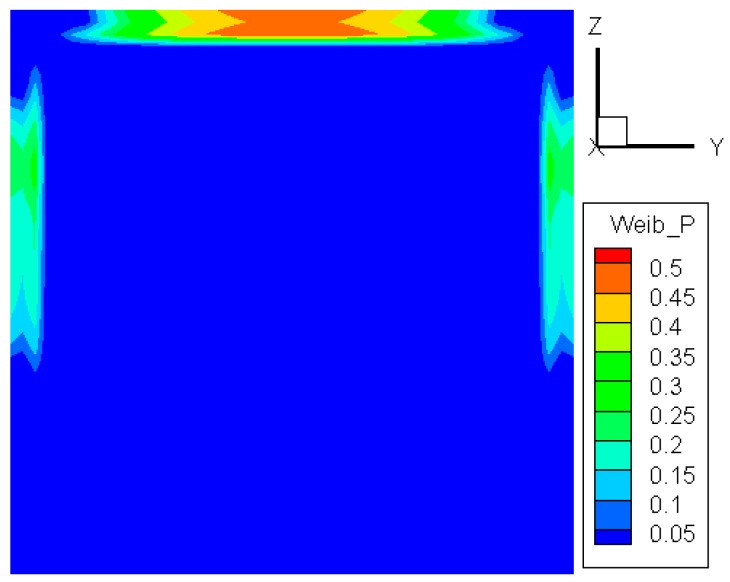
Probability distribution of crack initiation via the three-parameter Weibull distribution.

The three-parameter Weibull distribution is more robust and may provide a better characterization of the data [19]. By setting the same simulation condition, the probability distribution of the three-parameter model is obtained and shown in Figure 4. It can be seen that the contour plot is similar to that of the two-parameter model. A slightly different feature is that the highest value now appears exactly in the central part of the upper edge. The values of the critical temperature difference for the first crack are predicted as 94 °C and 93 °C by the two- and three-parameter models, respectively. The results exhibit good coincidence and are close to the value of 90 °C reported by Skelly [5].

### 3.2. Deterministic Criterion

Another way to predict the crack initiation is to use a deterministic criterion. To perform this, we employ the following five strength theories:

#### 3.2.1. Maximum Normal Stress Criterion

**Figure 5:**
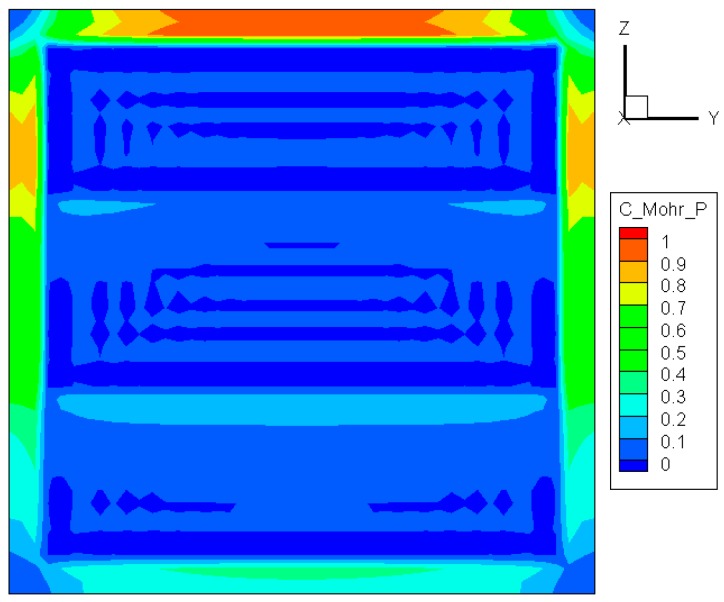
Distribution of $\max(\sigma_{1},\sigma_{2},\sigma_{3})/S_{\mathrm{ut}}$ before crack initiation.

The maximum normal stress criterion, also known as Coulomb’s criterion, states that a crack occurs when the maximum normal stress reaches the ultimate strength of the material [20]. The ultimate tensile strength is smaller than the compressive strength in most cases. We assume the same ultimate strength *S*_ut_ in tension and compression, which means that the crack forms in the multi-axial state of stress, when the maximum principal normal stress exceeds the ultimate tensile or compressive strength. This is the safer way to predict the crack initiation, and can be written as:

(5) $$\max(\sigma_{1},\sigma_{2},\sigma_{3})\geq S_{\mathrm{ut}}$$

where *S*_ut_ is the ultimate tensile stress of the glass pane. Here *S*_ut_ was taken as 40 MPa for the float glass without consideration of flaw distribution. The distribution of $\max(\sigma_{1},\sigma_{2},\sigma_{3})/S_{\mathrm{ut}}$ is plotted in Figure 5. Similar to the results given by the probabilistic criterion, the maximum value is seen at the center of the upper edge. At the center of the glass pane, there are some grades which are not visible in Figure 3 and Figure 4. These differences are caused by the calculation methods.

#### 3.2.2. Coulomb-Mohr Criterion

**Figure 6:**
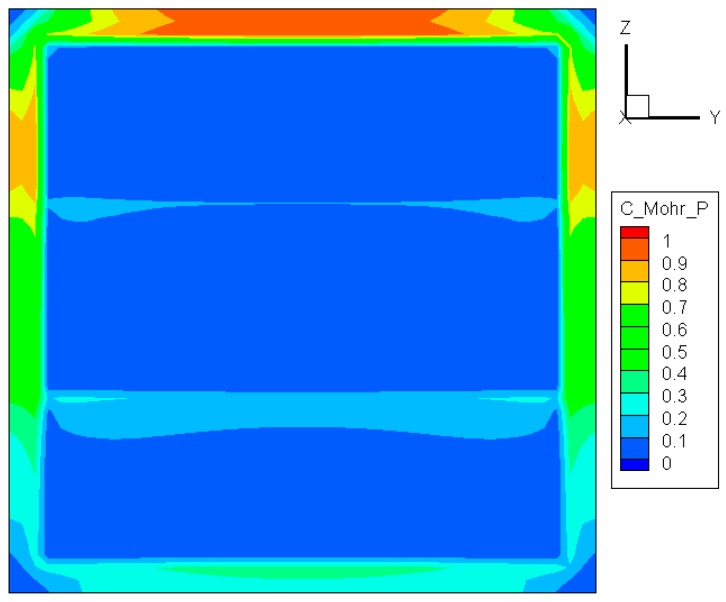
Distribution of $\sigma_{1}/S_{\mathrm{ut}}-\sigma_{3}/S_{\mathrm{uc}}$ before crack initiation.

The Coulomb-Mohr criterion asserts that fracture occurs when the principal stresses satisfy the following condition [21]:

(6) $$\frac{\sigma_{1}}{S_{\mathrm{ut}}}-\frac{\sigma_{3}}{S_{\mathrm{uc}}}\geq 1$$

with *S*_ut_ and *S*_uc_ being the ultimate tensile and compressive strengths, respectively. Note that both *σ*_3_ and *S*_uc_ are always negative. As can be noted in Figure 6, the maximum value of $\sigma_{1}/S_{\mathrm{ut}}-\sigma_{3}/S_{\mathrm{uc}}$ is located at the center of the upper edge, meaning that the cracking of the glass initiates from that area. This case is consistent with the foregoing predictions. Considerable differences between Figure 5 and Figure 6 appear in the exposed part of the pane, but have no significant effect on crack initiation because the stress there is much smaller than that in the edge region.

#### 3.2.3. Maximum Principal Stress Criterion

The maximum principal stress criterion can be expressed by:

(7) $$\frac{\sigma_{1}}{S_{\mathrm{ut}}}\geq 1$$

**Figure 7:**
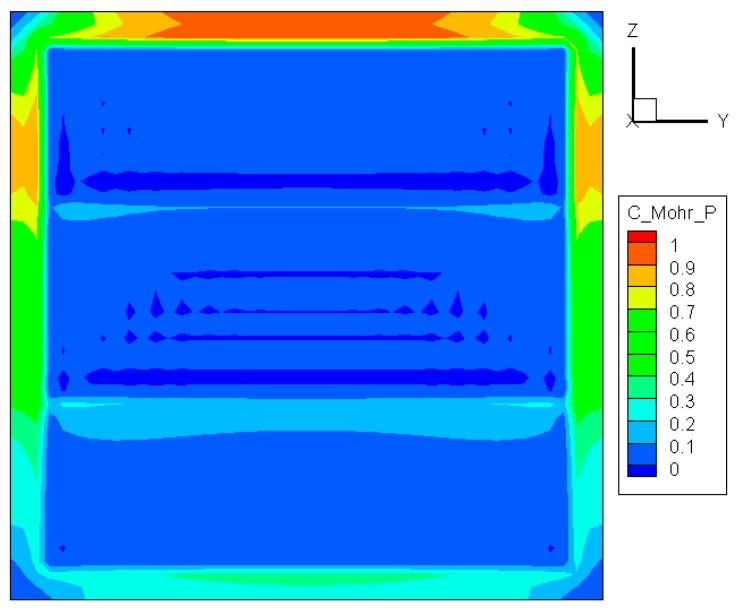
Distribution of $\sigma_{1}/S_{\mathrm{ut}}$ before crack initiation.

This means that the crack occurs when the largest principal stress *σ*_1_ exceeds the uniaxial tensile strength. Although this criterion allows for a quick and simple comparison with experimental data, it is rarely suitable for design purposes [22]. The distribution of $\sigma_{1}/S_{\mathrm{ut}}$ is depicted in Figure 7. As in Figure 5 and Figure 6, the maximum value is also located at the top center of the glass edge. At the same time, it is interesting to see that the distribution in the exposed region of the glass resembles a combination of those shown in Figure 5 and Figure 6. Since the only involved principle stress *σ*_1_ is always positive, this criterion can be used only if the glass is under expansion.

#### 3.2.4. Maximum Mises Stress Criterion

Also known as the shear-energy theory or the maximum distortion energy theory, this criterion states that a material starts to crack at a location where the von Mises stress attains the yield strength [23]:

(8) $$\frac{\sigma_{\mathrm{vm}}}{0.577S_{\mathrm{ut}}}\geq 1$$

**Figure 8:**
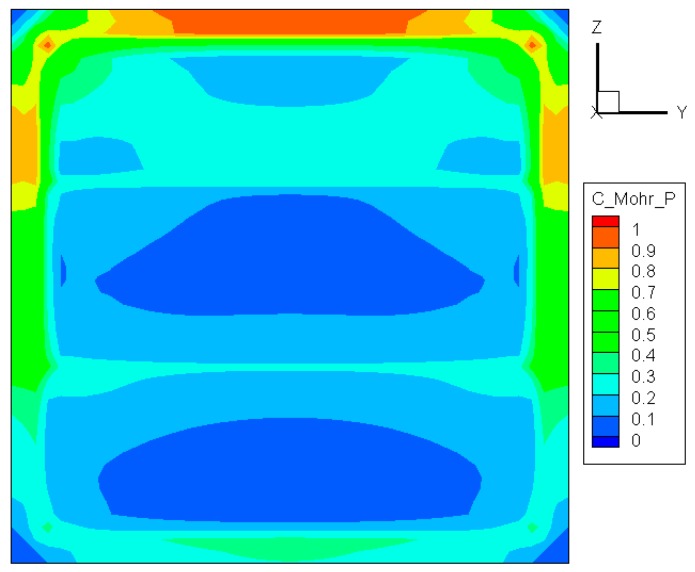
Distribution of $\sigma_{\mathrm{vm}}/0.577S_{\mathrm{ut}}$ before crack initiation.

As shown in Figure 8, the maximum value of $\sigma_{\mathrm{vm}}/0.577S_{\mathrm{ut}}$ appears at the top center, in accordance with the results given by the other criteria mentioned above. However, clear gradation can be seen in the three regions where the temperatures are different due to unequal heating rates. In all three regions, the lowest Mises stress occurs at the central part, implying that it is difficult for a crack to initiate there.

#### 3.2.5. Maximum Shear Stress Criterion

The maximum shear stress criterion is also known as the Tresca yield criterion, and it predicts failure of a material when the absolute maximum of the shear stress reaches the strength of simple tension [23], i.e.,

(9) $$\frac{\max(\sigma_{xy},\sigma_{xz},\sigma_{yz})}{0.5S_{\mathrm{ut}}}\geq 1$$

**Figure 9:**
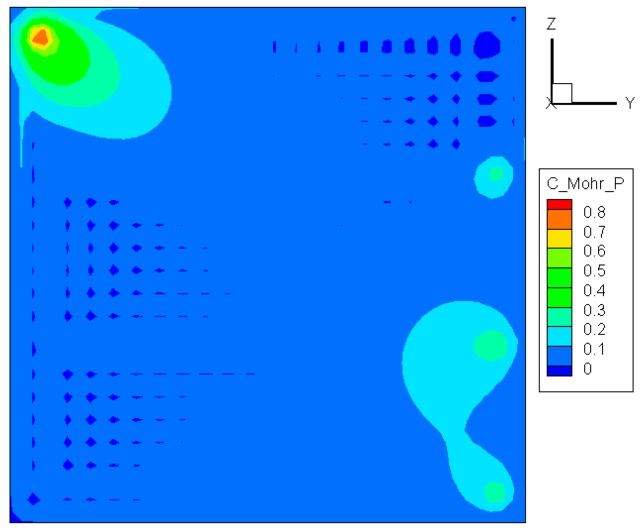
Distribution of $\max(\sigma_{xy},\sigma_{xz},\sigma_{yz})/0.5S_{\mathrm{ut}}$ before crack initiation.

The distribution of $\max(\sigma_{xy},\sigma_{xz},\sigma_{yz})/0.5S_{\mathrm{ut}}$ is shown Figure 9. The pattern is dramatically different from those obtained from any other criteria, and the largest value appears near the top-left corner. However, this does not mean that cracks will initiate from that position as well, because under the thermal loading the normal stresses are much larger than the shear stress. Therefore, this criterion cannot be used to predict crack initiation as it excludes the contribution from normal stresses.

All the methods mentioned above were applied to examine the time to first crack, and the results are listed in Table 2. For the reasons demonstrated above, no crack appears during the period of simulation when the maximum shear stress criterion is used. This exception aside, one can see that the deterministic methods No. 3 to 6 give shorter times to first crack than the probabilistic methods No. 1 to 2. Particularly, the Maximum normal stress, Coulomb-Mohr, and Maximum principle criteria lead to an identical result of 23.5 s. This is the shortest time to first crack and can be used as a criterion for crack initiation. In comparison, the maximum von Mises stress criterion results in a slightly longer time of 25 s.

## 4. Modeling of Crack Propagation

**Figure 10:**
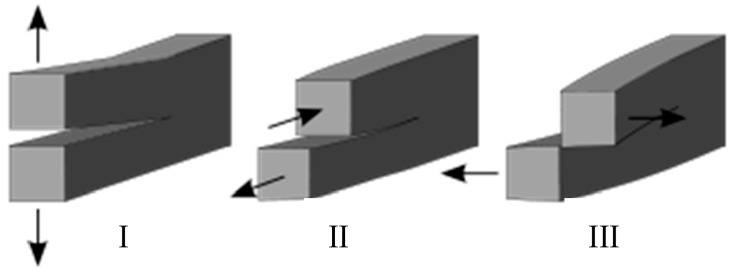
Crack Mode I, Mode II, and Mode III.

Three linearly independent cracking modes are categorized as Mode I, II, and III, as shown in Figure 10. Mode I is an opening mode where the crack surfaces move directly apart and it is the most common load type encountered in engineering design. Mode II is a sliding mode where the crack surfaces slide over one another in a direction perpendicular to the leading edge of the crack. Mode III is a tearing mode where the crack surfaces move relative to one another and parallel to the leading edge of the crack. Different subscripts are used to designate the stress intensity factor (*K*) and the energy release rate (*G*) for the three different modes. The *K* and *G* for mode I are designated *K*_I_ and *G*_I_ when applied to the crack opening mode. The mode II stress intensity factor and energy release rate, *K*_II_ and *G*_II_, are applied to the crack sliding mode, and the mode III stress intensity factor and energy release rate, *K*_III_ and *G*_III_, are applied to the tearing mode.

Numerical simulation of crack propagation has been a challenging problem for many years, and discrete crack propagation modeling approaches using remeshing techniques are usually adopted. The moving tip remesh method is utilized here to refine the tip field of the crack. The following six criteria are chosen to determine whether a crack grows or is arrested.

### 4.1. Mixed-Mode Criterion Based on SIFs

The SIFs-based mixed-mode criterion is commonly used to predict crack growth. It assumes that cracks start to grow once the stress intensity factors *K*_I_, *K*_II_, and *K*_III_ satisfy the following condition [24]:

(10) $${\left(\frac{K_{I}}{K_{\mathrm{IC}}}\right)}^{\alpha }+{\left(\frac{K_{\mathrm{II}}}{K_{\mathrm{IIC}}}\right)}^{\beta }+{\left(\frac{K_{\mathrm{III}}}{K_{\mathrm{IIIC}}}\right)}^{\gamma }=1$$

where *K*_IC_, *K*_IIC_, and *K*_IIIC_ denote the values of fracture toughness of the I, II and III modes, respectively, and *α*, *β*, and *γ* are empirical constants. Choosing α=β=2, and ignoring the term of the tearing mode *γ*, as suggested by Wu [25], yields

(11) $${\left(\frac{K_{I}}{K_{\mathrm{IC}}}\right)}^{2}+{\left(\frac{K_{\mathrm{II}}}{K_{\mathrm{IIC}}}\right)}^{2}=1$$

**Figure 11:**
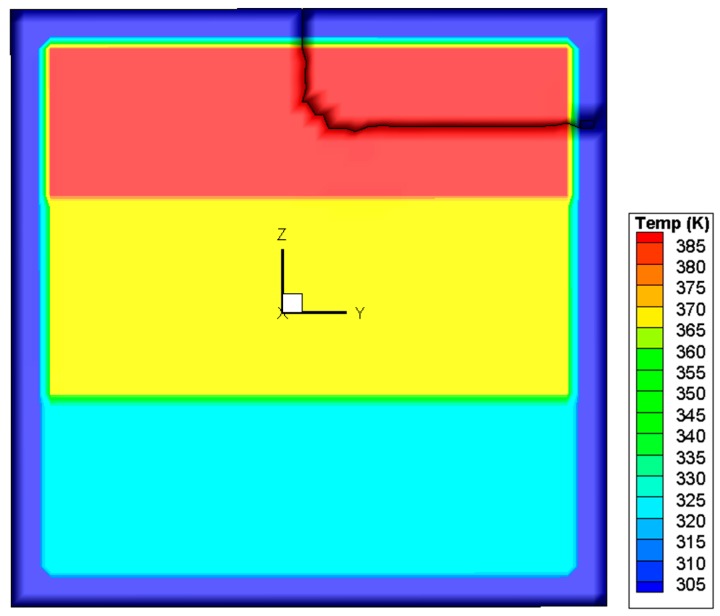
Crack path predicted by the mixed-mode criterion based on SIFs.

**Figure 12:**
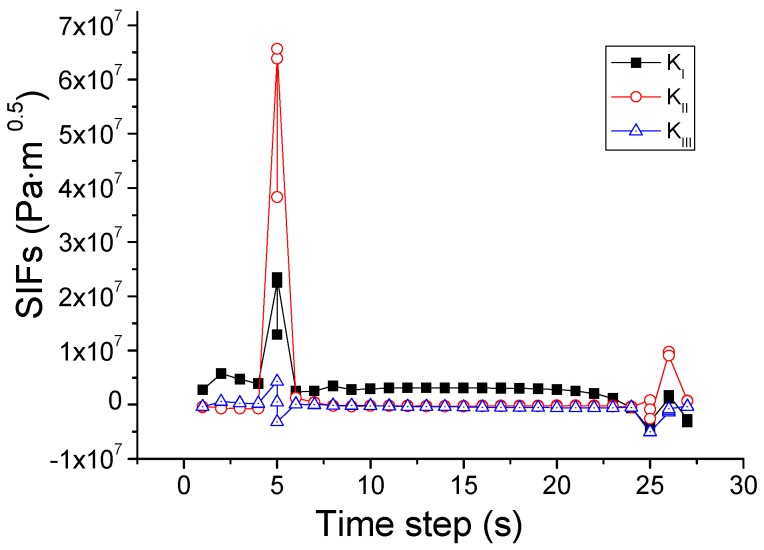
Variation of SIFs associated with the mixed-mode criterion based on SIFs.

Based on this criterion, the crack propagation path is predicted and shown in Figure 11. The crack starts from the center of the upper edge, and then propagates downwards to the center of the upper region in the pane. During this process, as illustrated in Figure 12, *K*_I_ is initially larger than *K*_II_ and *K*_III_ and thus dominates the crack direction. Thereafter, *K*_II_ becomes larger than *K*_I_, so the crack changes the propagation direction to the right of the glass.

### 4.2. Mixed-Mode Criterion Based on Energy Release Rates

In this case, cracks are assumed to grow if the energy release rates *G*_I_, *G*_II_, and *G*_III_ satisfy [24] the following condition:

(12) $${\left(\frac{G_{I}}{G_{\mathrm{IC}}}\right)}^{\alpha }+{\left(\frac{G_{\mathrm{II}}}{G_{\mathrm{IIC}}}\right)}^{\beta }+{\left(\frac{G_{\mathrm{III}}}{G_{\mathrm{IIIC}}}\right)}^{\gamma }=1$$

where *G*_IC_, *G*_IIC_, and *G*_IIIC_ are the critical energy release rates in the I, II and III modes, respectively, and *α*, *β*, and *γ* are constants. By setting *G*_Ic_ = *G*_IIc_ = *G*_IIIc_ = *G*_c_ and α=β=γ=1, the above condition is reduced to the fracture criterion based on the total energy release rate [26]:

(13) $$G=G_{I}+G_{\mathrm{II}}+G_{\mathrm{III}}=G_{c}$$

**Figure 13:**
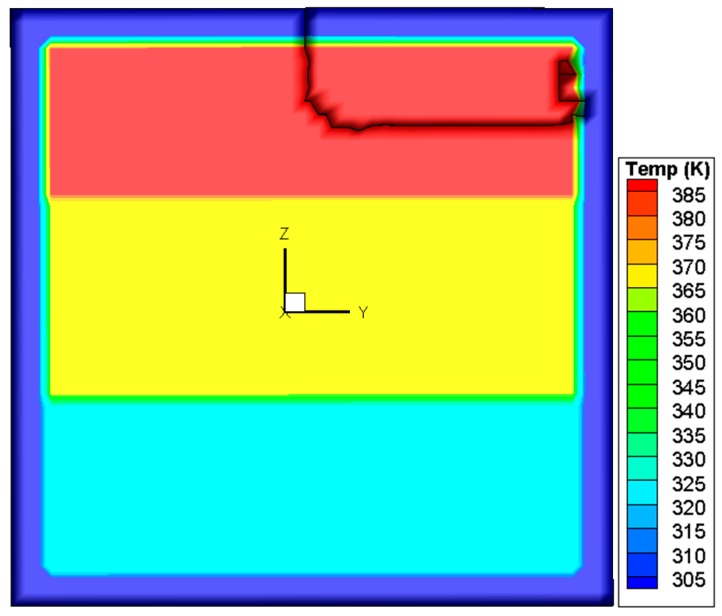
Crack path predicted by the mixed-mode criterion based on energy release rates.

**Figure 14:**
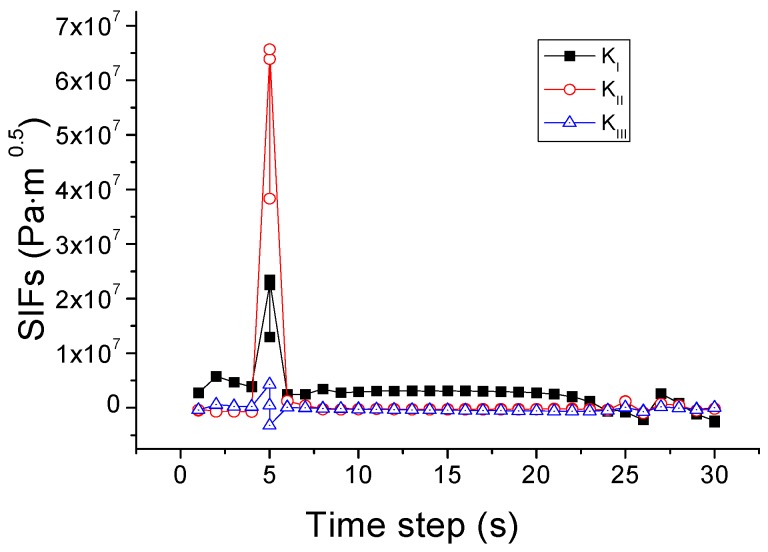
Variation of SIFs associated with the mixed-mode criterion based on energy release rates.

**Figure 15:**
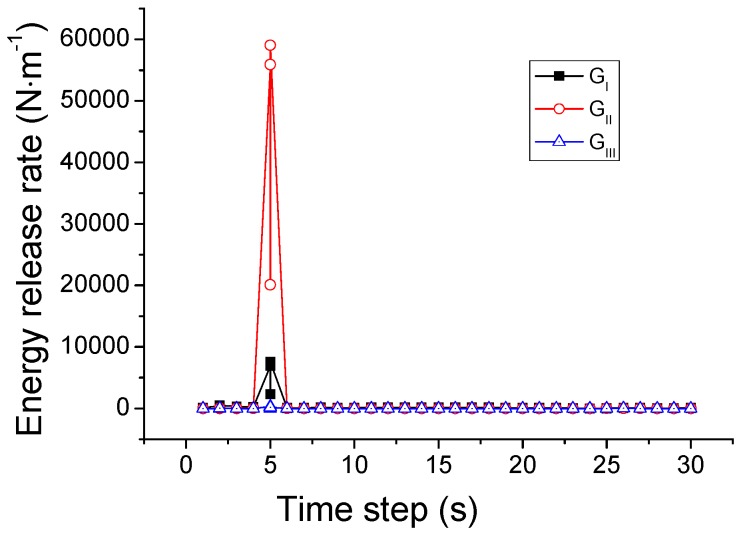
Variation of energy release rates associated with the mixed-mode criterion based on energy release rates.

This criterion is different from the SIFs-based mixed-mode criterion in the power value of *α*, *β*, and *γ*. The *K*_III_ is ignored in the SIFs-based mixed-mode criterion and *G*_III_ is considered in the energy release rates based mixed-mode criterion. The simulated crack path using this criterion is plotted in Figure 13. In comparison with the case of the SIF criterion, a difference is that the crack changes its direction once again when it reaches the right edge of the pane and then propagates upwards. For elucidation, the variations of the SIFs and energy release rates during the whole process are depicted in Figure 14 and Figure 15. It is seen that the growth of the crack is first dominated by *K*_II_ or *G*_II_, and then by *K*_I_ or *G*_I_, as in the situation shown in Figure 12. However, in the last stage when the crack reaches the right edge of the glass, the values of *K*_II_ and *K*_I_ come very close to each other, and neither can dominate the crack direction. A possible result in this circumstance is the vertical crack propagation, because the horizontal path is constrained by the protecting frame.

### 4.3. SIF-Based Maximum Circumferential Stress Criterion

**Figure 16:**
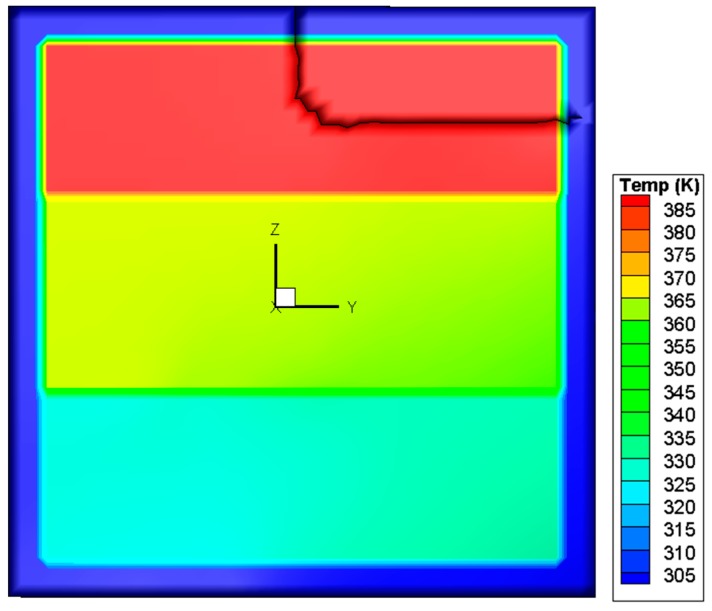
Crack path predicted by the SIF-based maximum circumferential stress criterion.

**Figure 17:**
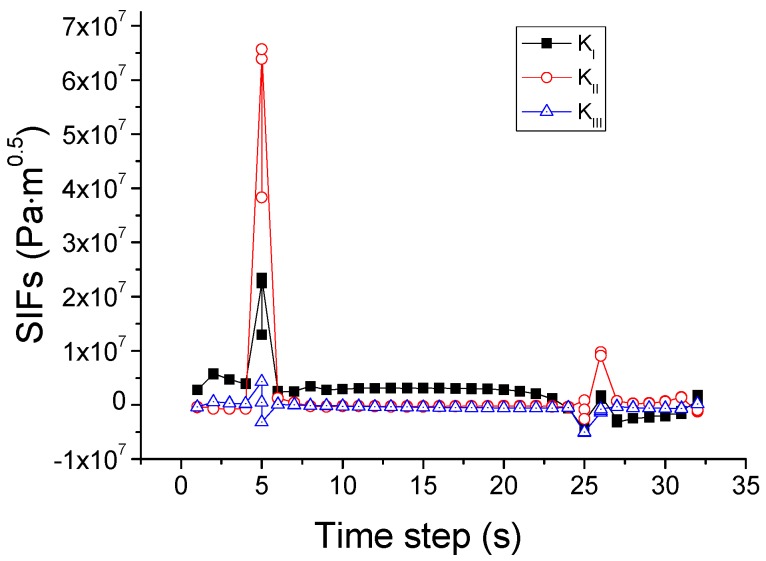
Variation of SIFs associated with the maximum circumferential stress criterion.

This criterion states that a crack grows in the direction of the maximum circumferential stress [24,27] which is determined by:

(14) $$K_{e}=\cos\frac{\theta_{0}}{2}(K_{\mathrm{Ieff}}\;\cos^{2}\frac{\theta_{0}}{2}-\frac{3}{2}K_{II}\sin\theta_{0})=K_{\mathrm{IC}}$$

(15) $$K_{\mathrm{Ieff}}=K_{I}+B|K_{\mathrm{III}}|$$

where *B* is an empirical factor, *K*_e_ is the effective stress intensity factor, *K*_IC_ is the fracture toughness, and *θ*_0_ is the angle of crack growth. According to Equations (14) and (15), the simulated crack path is shown in Figure 16. Again, a significant difference can only be found at the later stage of crack propagation: the crack is arrested once it runs into the edge region protected by the frame. This phenomenon can be understood with the aid of Figure 17 in which the variations of the SIFs are given. Although *K*_II_ is larger than *K*_I_ at that stage, it is not sufficient to drive further growth of the crack arriving at the edge region.

### 4.4. Criterion Based on Maximum Principal Stress

**Figure 18:**
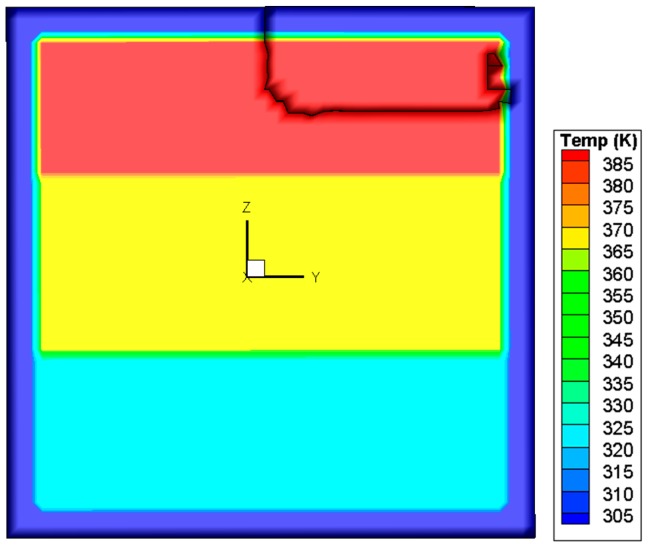
Crack path predicted by the criterion based on maximum principal stress.

**Figure 19:**
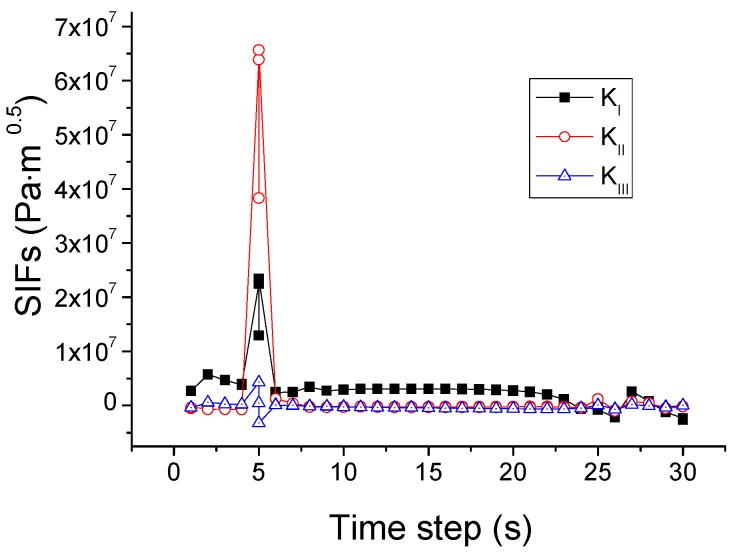
Variation of SIFs associated with the criterion based on maximum principal stress.

This criterion states that a crack grows in the direction where the maximum principal stress exceeds the tensile strength of the material. The implementation relies on a detailed stress distribution and thus requires very fine meshes in the vicinity of the crack tip in the finite element simulation. Figure 18 shows the propagation path of the crack. The result is almost the same as that predicted via the mixed-mode criterion based on the energy release rate. The corresponding variation of the SIF, plotted in Figure 19, is almost the same as that given in Figure 14 as well. Therefore it can be concluded that both criteria result in almost the same path of crack propagation.

### 4.5. Criterion Based on Crack Tip Opening Angle (CTOA)

**Figure 20:**
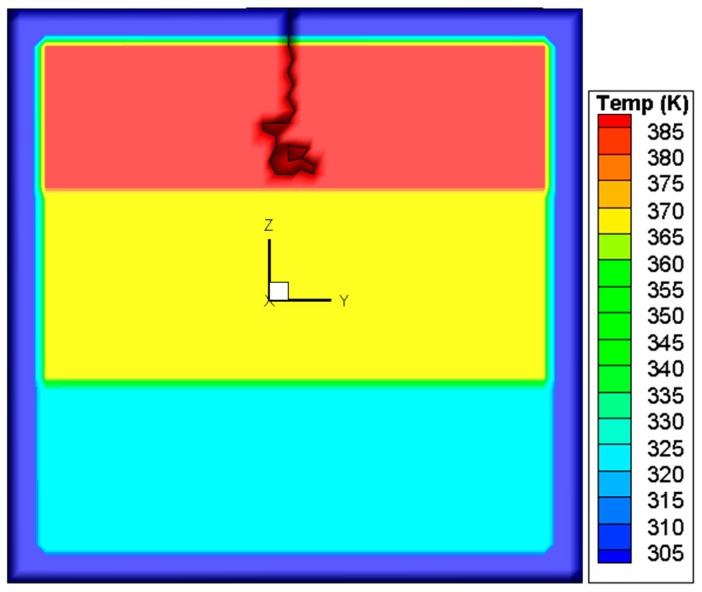
Crack path predicted by the CTOA-based crack growth criterion.

**Figure 21:**
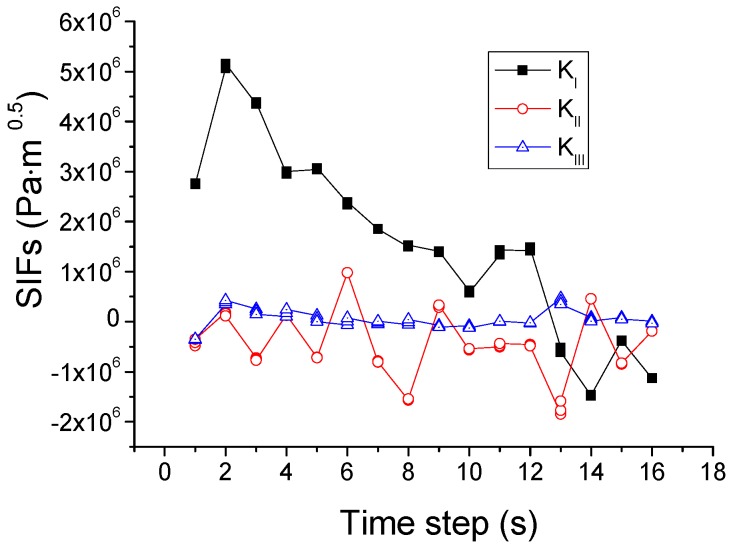
Variation of SIFs associated with the CTOA-based crack growth criterion.

According to this criterion, the crack is extended if the CTOA exceeds a critical value [28]. The evaluation of the CTOA is based on the angles of sub-cracks and in the simulation of uniform crack growth, and the actual 3D crack angle is considered as the average of all the sub-crack angles. By setting the critical CTOA value as 0.00001 for the glass [29], the crack path is obtained and shown in Figure 20. It is visible that the crack starts from the center of the upper edge, then runs downwards to the center of the upper region with higher temperature. After some frequent but short-lived transitions in the direction, the crack goes back before reaching the boundary between the upper and middle regions, finally forming an island in the upper region. The variations of the associated SIFs are provided in Figure 21. It can be seen that *K*_I_ tends to decrease with the crack growth, and *K*_II_ exhibits considerable fluctuation, while *K*_III_ remains nearly constant. In the initial stage *K*_I_ is larger than *K*_II_ and *K*_III_, thus dominating the crack growth. After about 13 time steps *K*_I_ becomes smaller, and *K*_II_ and *K*_III_ start to dominate the growth alternatively. This explains why the crack changes its direction frequently and leaves behind a closed trajectory to form an island.

### 4.6. Criterion Based on Crack Tip Opening Displacement (CTOD)

**Figure 22:**
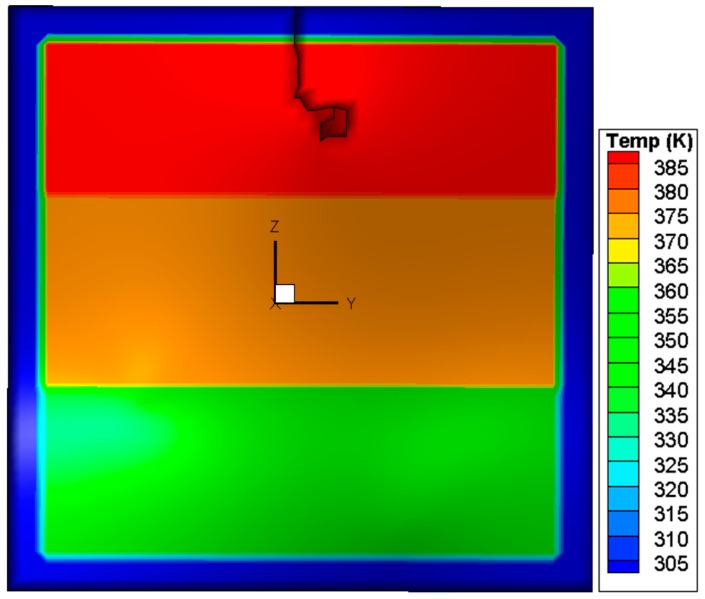
Crack path predicted by the CTOD-based crack growth criterion.

**Figure 23:**
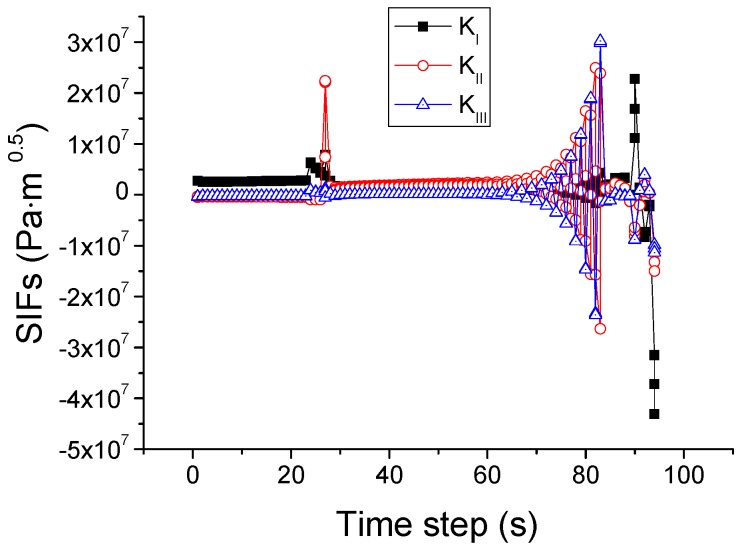
Variation of SIFs associated with the CTOD-based crack growth criterion.

This criterion postulates the growth of a crack when the current CTOD reaches a critical value, along the direction that causes either the opening or the shearing component of the CTOD at the new crack tip to be the absolute maximum. In the simulation of uniform crack growth, the actual 3D crack displacement is considered as the average of those for all the sub-crack angles [30]. L.C.S. Nunes and J.M.L. Reis [31] experimentally studied the CTOD and crack extension of glass using the digital image correlation method. It was found that the glass fiber starts to extend when the CTOD is larger than 0.00001 m. Based on this work, the critical CTOD value is taken as 0.00001 m to predict the glass extension here. The computed crack path is shown in Figure 22, with the corresponding variations in the SIFs given in Figure 23. The crack initiates at the top center of the glass, and propagates down towards the center of the upper region. After *K*_II_ becomes larger than *K*_I_, the crack turns to the right and is then arrested when the CTOD becomes less than 0.00001 m. From that time on, large fluctuations of the SIFs occur and the magnitude of *K*_I_ is always smaller than those of *K*_II_ and *K*_III_. Thus the direction of the crack is changed, leading to the formation of an island at the center of the upper region. When the CTOD is larger than 0.00001 m, such as 0.00002 m and 0.0001 m, the simulation results show that the crack no longer grows anymore under the same thermal loading condition. When the CTOD is smaller than 0.00001 m, such as 0.000005 m, the crack also grows. However, by comparing this with the other cases and Nune’s work [31], 0.000005 m is not suitable to predict the crack growth.

### 4.7. Discussion

**Figure 24:**
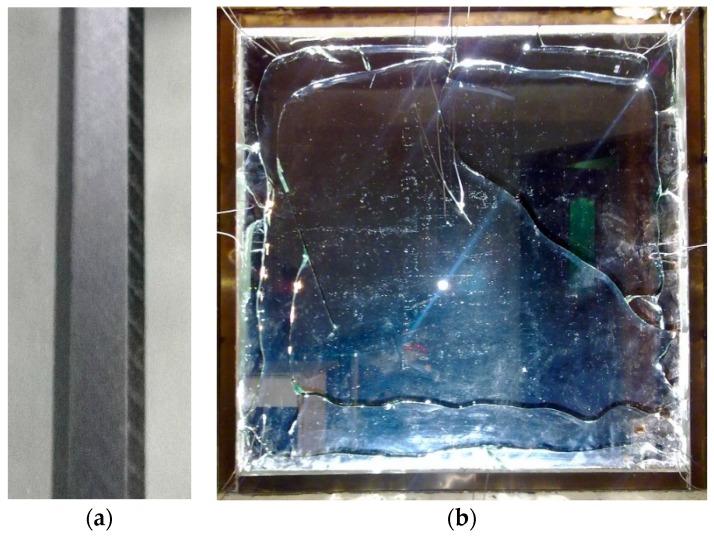
The (**a**) edge condition and (**b**) a typical crack pattern in a glass pane under thermal loading.

Several criteria for crack initiation and propagation are examined in the simulation. It should be noted that in a fire the heat transfer between glass and smoke or fire is very complicated, and includes radiation and convention, and the interaction of the fire and glass may markedly change the enclosure fire dynamic. These factors as well as the inherent characteristics in brittle materials, such as microcracks [32], may cause the crack initiation to be very random. On the other hand, from previous experiments it was found that despite the tests that were conducted under nearly identical conditions, the critical breakage stresses distributed randomly [4]. Thus, uncertainty exists in crack initiation and when considering the fire scenario and glass properties, it is difficult to predict glass breakage occurrence very accurately. Regarding the crack path, according to the numerical results there are essentially two kinds of crack path. The first is the crack path that starts from the central upper edge and then terminates after turning nearly a right-angle at the right edge of the upper region, as predicted by using the criteria based on SIFs, energy release rates, and maximum principal stresses, as well as the SIF-based maximum circumferential stress criterion. The other is the crack path initiating from the upper center of the pane edge and stopping in the central part of the upper region with the formation of an island, as predicted by the CTOA- and CTOD-based crack growth criteria. If the conditions in the experiments are controlled very strictly to generate the crack path, the phenomenon in crack propagation, such as oscillatory behavior, may be deterministic rather than a statistical process [33,34], while also due to the microcrack and thermal gradient, the crack path presents probabilistic characteristics in a fire. For comparison, a typical experimental result of the crack path in a heated glass pane that was observed in our previous study [35] is shown in Figure 24. The glass critical strain decreases with increasing microcrack size, so the edge of the glass has been polished to avoid the strength reduction. The experimental condition is similar to that used in the simulation, and for more information please refer to [35]. The crack in Figure 24 initiates from the top center of the glass, and propagates along the upper edge to the right and left. Relatively, the situation seems more consistent with the first kind of crack path in the simulation. It is therefore believed that the criteria based on SIFs, energy release rates, and maximum principal stresses, as well as the SIF-based maximum circumferential stress criterion, are more suitable for use in predicting crack growth in glazing exposed to a fire, rather than the CTOA- and CTOD-based criteria. Unfortunately, the program used here can currently only model one crack propagation, and further effort is needed to develop a code which can simulate the complicated process of crack initiation and growth in a real fire scenario.

## 5. Conclusions

Due to the stochastic nature, prediction of fracture and fall-out of window glasses exposed to fire is still an unsolved problem. In an effort to solve this, crack initiation and propagation in a model glass pane under a fire condition has been investigated using the finite element method. Both the probabilistic and deterministic methods were adopted to examine crack initiation, while several commonly used criteria were utilized to predict crack propagation. Through detailed data analysis and comparison with the experimental observations, the following conclusions can be made.

The two- and three-parameter Weibull distributions give almost the same location and temperature difference for crack initiation. However, the predicted critical temperature difference is much larger than that determined via the criteria of maximum tensile stress, maximum normal stress, and maximum Mises stress, as well as the Coulomb-Mohr criterion. These last four criteria can be used to model the initiation of the crack in the glass pane. In contrast, the maximum shear stress criterion predicts no crack initiation and thus should be abandoned.

The mixed-mode criteria based on SIF, energy release rate, and the maximum principal stress, as well as the SIF-based maximum circumferential stress criterion, can provide relatively consistent crack propagation paths with the experiment. At the same time, the predictions via the CTOA- and CTOD-based crack growth criteria show considerable differences. Consequently, the first four criteria are more suitable for modeling the crack propagation.

The actual case of glass crack in fire is very complex, and includes bifurcations and multi-cracks. The fracture theory and technology to simulate such a complicated phenomenon are still under development. At present, only one crack is simulated, and the simulation of multi-cracks will have to be performed in the future. Once the multi-crack can be simulated, it will be easier to compare the simulated results with experimental results.

## Figures and Tables

**Table 1:** Parameters for two- and three-parameter Weibull Distribution Function (4).

Weibull Function	*m*	*σ*_0_ (MPa)	*σ*_u_ (MPa)
Two-parameter	3.20	74.1	–
Three-parameter	1.21	33.0	35.8

**Table 2:** Time to first crack predicted by various criteria.

No.	Criteria	ΔT, °C	*σ_y_*, MPa	*σ*_vm_, MPa	*σ*_1_, MPa	Time, s
1	Two-parameter Weibull	94	67.78	67.23	67.79	44.0
2	Three-parameter Weibull	93	60.50	59.80	60.50	43.0
3	Maximum normal stress	76.8	40.03	39.48	40.03	23.5
4	Coulomb-Mohr	76.8	40.03	39.48	40.03	23.5
5	Maximum principle stress	76.8	40.03	39.48	40.03	23.5
6	Maximum von Mises stress	78.0	40.72	40.14	40.73	25.0
7	Maximum shear stress	no crack	–	–	–	–
